# Synthesis of adenosine analogues with indole moiety as human adenosine A_3_ receptor ligands

**DOI:** 10.1098/rsos.171596

**Published:** 2018-02-07

**Authors:** Yan Xia, Xiliang Zheng, Erkang Wang, Dongfeng Li, Ruibin Hou, Jin Wang

**Affiliations:** 1 State Key Laboratory of Electroanalytical Chemistry, Changchun Institute of Applied Chemistry Chinese Academy of Sciences, Renmen Street, Changchun 130022, People's Republic of China; 2 College of Chemistry and Life Science, Changchun University of Technology, Yanan Street No. 2055, Changchun 130012, People's Republic of China; 3 Advanced Institute of Materials Science, Changchun University of Technology, Yanan Street No. 2055, Changchun 130012, People's Republic of China; 4 Department of Chemistry and Physics, State University of New York at Stony Brook, New York, NY, USA

**Keywords:** adenosine receptors, affinity, radioligand, A_3_AR modulation, molecular docking

## Abstract

Adenosine is an endogenous modulator exerting its functions through the activation of four adenosine receptor (AR) subtypes, termed A_1_, A_2A_, A_2B_ and A_3_, which belong to the G-protein-coupled receptor superfamily. The human A_3_AR (hA_3_AR) subtype is implicated in several cytoprotective functions. Therefore, hA_3_AR modulators, and in particular agonists, are sought for their potential application as anti-inflammatory, anti-cancer and cardioprotective agents. Here, we prepared novel adenosine derivatives with indole moiety as hA_3_AR ligands. According to the biological assay, we found that 2-substituents **11** were critical structural determinants for A_3_AR ligands (*K*_i_ = 111 nM). The observed structure–affinity relationships of this class of ligands were also exhaustively rationalized using the molecular modelling approach. This allows the investigation on the binding mode of the potential compound in the ligand-binding pocket of the human A_3_ receptor. The results demonstrated that **11** can interact with the ASN250, GLN167, PHE168 and VAL178 through hydrogen bonding, which are shown to be important for ligand–receptor interaction.

## 1. Introduction

Adenosine is an endogenous purine nucleoside that modulates many physiological processes, which is composed of a molecule of adenine attached to a ribose sugar molecule (ribofuranose) moiety via a β-*N*_9_-glycosidic bond [1–3]. Adenosine is widely found in nature and plays an important role in biochemical processes, such as energy transfer as adenosine triphosphate (ATP) and adenosine diphosphate (ADP), as well as in signal transduction as cyclic adenosine monophosphate (cAMP). It is also a neuromodulator, believed to play a role in promoting sleep and suppressing arousal. Adenosine also plays a role in the regulation of blood flow to various organs through vasodilation [4–6]. Cellular signalling by adenosine occurs through four known adenosine receptor (AR) subtypes (A_1_, A_2A_, A_2B_ and A_3_) [7]. All AR subtypes are G-protein-coupled receptors. The four receptor subtypes are further classified based on their ability to either stimulate or inhibit adenylate cyclase activity. They have long been considered to be promising therapeutic targets in a wide range of conditions, ranging from cerebral diseases to cancer, including inflammatory and immunological disorders [8,9]. Adenosine contributes in a significant manner to the maintenance of tissue integrity by modulating the immune system. Encouraging results have emerged with AR ligands for the management of several physiological conditions in preclinical and clinical settings [7,10].

Among the four AR subtypes, the A_3_AR, probably the most studied AR subtype, is also ubiquitously expressed [11]. The distribution of A_3_AR is species-dependent, and in humans, this subtype is expressed in the lungs, liver, heart, kidneys and brain [12–14]. The widespread distribution in different cells and tissues of the A_3_AR suggests a potential involvement in various pathologies and the possible use as a selective pharmacological target [15]. This subtype of ARs is involved in a variety of important pathophysiological processes, ranging from modulation of cerebral and cardiac ischaemic damage to regulation of immunosuppression and inflammation [16].

The increasing knowledge about A_3_ARs, in particular regarding the molecular biology of this subtype, has provided important evidence to consider this receptor as a novel therapeutic target. In addition, it enables rational design and the development of potent and selective A_3_AR ligands as promising therapeutic options for a variety of diseases [17,18]. Therefore, small molecule modulators targeting the A_3_AR have been sought for their potential application as anti-inflammatory, anti-cancer and cardioprotective agents [19–21].

Indoles probably represent one of the most important structural classes in drug discovery [22]. The indole substructure is a basic element for a number of biologically active natural and synthetic products. Indoles are found in a wide range of therapeutically important drugs [23,24]. Over the years, a considerable amount of effort has been made to find indoles with various biological activities and select certain agents for leads in drug discovery research. On account of their potent biological activities, indoles have continued to attract the interest of chemists and biologists alike in drug discovery.

In addition, the scientific community is making intensive efforts to design AR ligands endowed with greater selectivity or to develop innovative compounds acting as receptor modulators. To further explore the importance of indole framework as the basis for AR ligands, we investigate the receptor subdomain that binds the purine moiety by the study of 2-*O*-alkyl derivatives of 2-chloroadenosine. The present paper reports on the synthesis and binding studies of these compounds as well as of adenosine. We describe the lengths of alkyl group in terms of potency at the A_3_ receptor as well as receptor subtype selectivity. Here, we use the conversion of the 2-alkynyl group into flexible 2-*O*-alkyl between purine and indole moiety.

## 2. Results and discussion

### 2.1. Synthesis

**Scheme 1.**
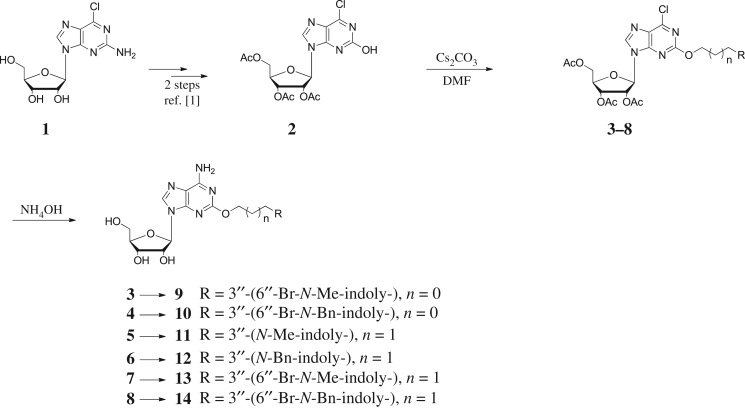
Synthesis of compounds **9–14**.

In an effort to discover new nucleoside analogues as potential A_3_AR ligands, we pursued the 2-oxypurine nucleoside using indole alkyl iodides as shown in scheme 1. The synthesis of the 5′-CH_2_OH analogues started from 2-amino-6-chloropurine riboside **1**, which was converted into 6-chloro-2-hydroxy-9-(2,3,5-tri-*O*-acetyl-β-d-ribofuranosyl)purine **2**, as reported [25]. The reaction of the hydroxyl group at the 2-position of **2** with various indole iodides was conducted in the presence of caesium carbonate to affect compounds **3–8**. Simultaneous removal of the acetyl group and amination at the 6-position of **3–8** using ammonium hydroxide solution yielded compounds **9–14**.

**Scheme 2.**
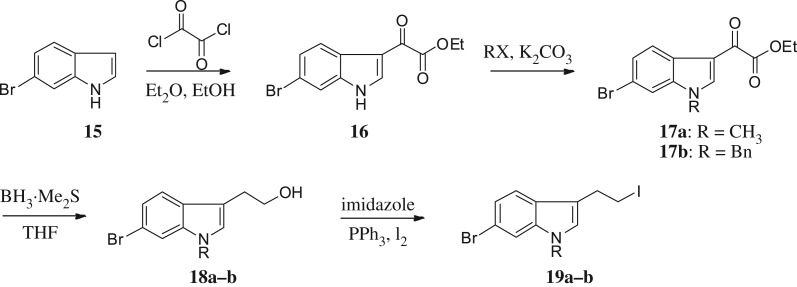
Synthesis of intermediates **19a–b**.

**Scheme 3.**
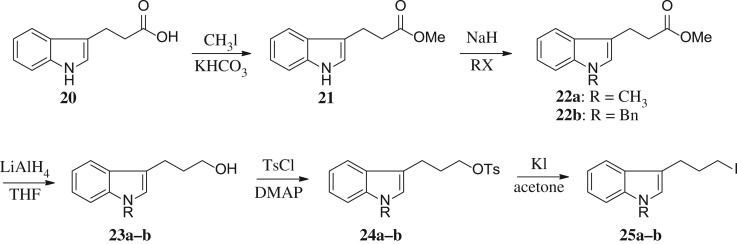
Synthesis of intermediates **25a–b**.

**Scheme 4.**
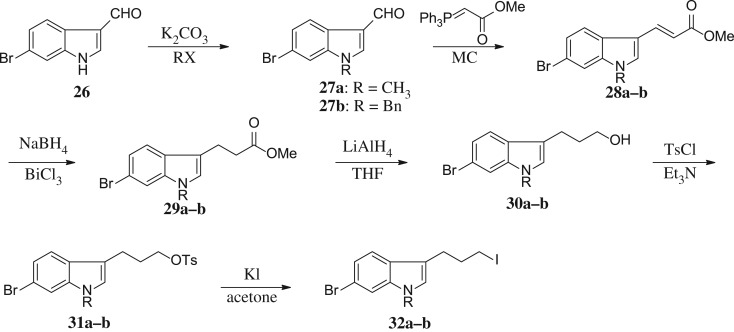
Synthesis of intermediates **32a–b**.

For synthesizing the key compounds, we should synthesize intermediates for the 2-ether component depicted in schemes 2–4. Scheme 2 illustrates the synthesis of two carbons as linker indole iodides. The indole 2-oxoacetate **16** was accessed by the treatment of 6-bromoindole **15** to oxalyl chloride, followed by a reaction with ethanol. Alkylation of indole nitrogen by CH_3_I and BnBr provided *N*-alkyl **17a** and **17b**, respectively. The following exposure to BH_3_-SMe_2_ provided corresponding alcohols **18a** and **18b**. The alcohols were transformed to corresponding iodides **19a–b** by iodine, triphenylphosphine and imidazole.

The three carbons used as the linker of indole iodides shown in scheme 3 were obtained from the commercially available 3-indolepropionic acid **20**. Propionate **21** was prepared by the conversion of carboxylic acid into methyl ester by treatment with CH_3_I and KHCO_3_. Subsequent N-alkylation of the indole nitrogen provided **22a** and **22b** followed by reduction of carboxylic methyl ester to produce the corresponding alcohols **23a** and **23b**. Finally, iodides **25a** and **25b** were derived from **23a–b** via tosylate alcohol, which underwent substitution with iodine.

The synthetic strategy for the preparation of substituted indole derivatives is described in scheme 4. The commercially available 4-bromoindole-3-carboxaldehyde **26** was reacted with CH_3_I and BnBr to give rise to N-alkylation **27a–b**, respectively. The Wittig reaction of aldehyde provided *trans*-indole acrylate **28a–b**. The following treatment with NaBH_4_ and BiCl_3_ yielded propionates **29a–b**. LiAlH_4_ reduction of the carboxylic ester produced intermediates **30a–b** followed by treatment with tosyl chloride to afford the corresponding **31a–b**, and the tosylate moiety was displaced with good yield with iodine to incorporate the iodo functionality. Detailed synthetic procedures including the yield of reactions and characteristic data can be found in the electronic supplementary material.

### 2.2. Binding affinity studies

The aim of the present study is to expand knowledge of the structure–activity relationships at the A_3_AR and at other subtypes, both in relation to binding affinity and intrinsic efficacy, of adenosine derivatives modified in the 2-position. A screening campaign to discover new scaffolds for A_3_AR inhibition yielded the moderate-potency lead **11** (table 1). Among 2-substituted derivatives, 2-ethers were more potent than the corresponding amines or thioethers [26]. The effect of bromine substitution of the phenyl ring was evaluated. This series of 5′-brom analogue showed a tendency towards increased *K*_i_ values with A_1_ and A_3_ARs, depending on the bulkiness of the bromine atom. Of these analogues, compound **10** was equipotent to **13** with A_1_AR. However, its selectivity to binding A_3_AR was improved. Unexpectedly, **14** was threefold less potent than **13** in binding to A_3_AR and was tolerated by A_2A_AR. Although the 5′-bromo derivative **13** was somewhat equipotent to **11** with A_3_AR, its selectivity was reduced.

**Table 1.** Potency of 2-alkoxyadenosine derivatives to bind human A_1_, A_2A_ and A_3_ARs expressed in CHO cells.^a^ 
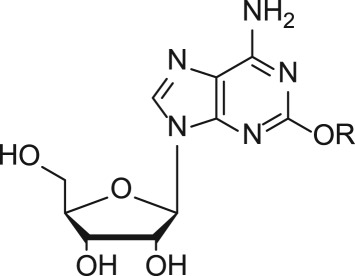

		*K*_i_ (nM ± SEM) or % inhibition at 10 µM		
entry	R	hA_1_AR^b^	hA_2A_AR^b^	hA_3_AR^b^
9	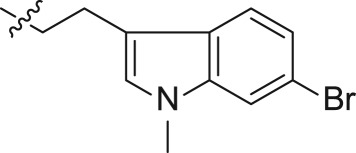	(47 ± 5%)	2770 ± 500	679 ± 149
10	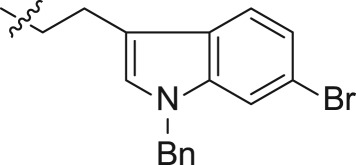	217 ± 57	380 ± 81	532 ± 144
11	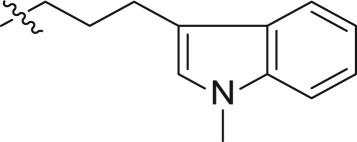	300 ± 70	880 ± 200	111 ± 30
12	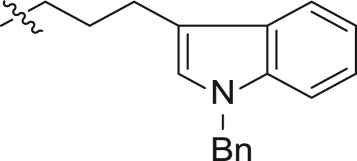	(34 ± 2%)	(56 ± 5%)	731 ± 209
13	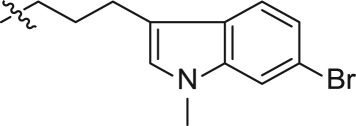	230 ± 26	262 ± 192	177 ± 43
14	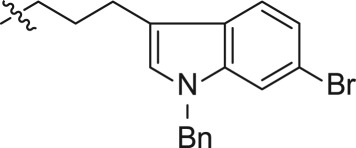	152 ± 49	371 ± 79	491 ± 143
	adenosine	—	—	290

^a^All experiments were performed on CHO cells stably expressing one of three subtypes of human ARs. The binding affinities for A_1_, A_2A_ and A_3_ARs were expressed as *K*_i_ values and were determined using agonist radioligands ([^3^H]CCPA), ([^3^H]CGS21680) and [^125^I]I-AB-MECA, respectively. Values in parentheses are for weak binding, corresponding to an IC_50_ ≥ 10 µM. Data are expressed as mean ± s.e.

^b^*K*_i_ in binding, unless noted.

Interestingly, the 3-indolyl analogue **11** was sevenfold more potent than **12** with A_3_AR. The increased size or steric hindrance of the *N*-substituent markedly decreases A_3_AR potency. Nevertheless, **12** was less potent than **13** in binding to A_2A_AR. The effect of the space of the alkyl chain between the 2-ethers and the indole moiety was tested. Elongation increased the affinity for A_3_AR. Compound **11** showed a fivefold increased potency with A_3_AR, while it was somewhat tolerated by A_2A_AR. The affinity of **9** to all three AR subtypes showed low potency compared with that of **13**, similar to the results with **10**.

However, the corresponding 2-indolyl derivative **13** was more potent than compound **11** in affinity for A_1_ and A_2A_Rs. The bulkiness of the *N*-substituent may be related to the increased affinity. Compound **14** was more potent than **12** in binding to all three ARs. Meanwhile, compound **11** displayed a fivefold potency enhancement over **9** with A_3_AR. The *N*-Bn derivative **10** with potency close to that of **9** was invariant in affinity for A_1_ and A_2A_Rs.

### 2.3. Molecular docking analysis

**Figure 1.**
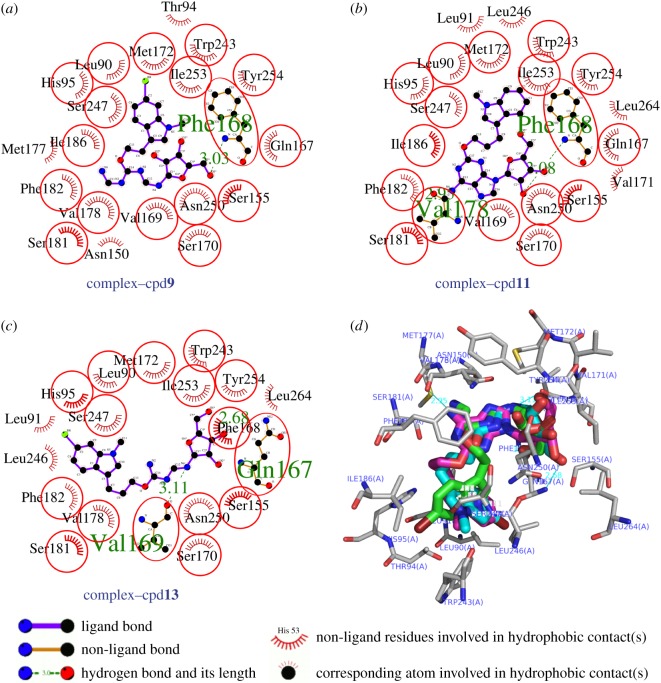
The two-dimensional image of ligand–hA_3_AR interactions with the hydrogen bonds and hydrophobic interactions (*a*–*c*); (*d*) the cpd**9** is coloured green, the cpd**11** is coloured magenta, the cpd**13** is coloured cyan, respectively. The surrounding residues interacting with these ligands are also shown and labelled. The meaning of the items on the two-dimensional plot above is listed in the bottom panel.

**Figure 2.**
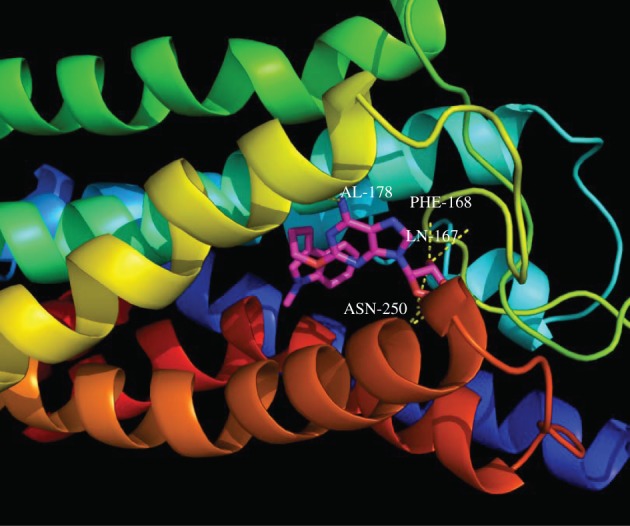
The most active compound cpd**11** (magenta colour) in the active site of human adenosine A_3_ receptor showing hydrogen bonding interactions.

Driven by docking of several derivatives with hA_3_R, we performed the molecular modelling studies [27] to explore the binding modes of all six aforementioned compounds as shown in table 2. Among these compounds, the compounds cpd**10**, **12** and **14** with the *N*-Bn substituent failed to dock into the ligand-binding site of the protein. The current docking results are consistent with the biological studies mentioned above and serve as an explanation for the sharply reduced affinity. On the contrary, all other three compounds (cpd**9**, **11** and **13**) with *N*-methyl substituent were found to dock into the binding pocket of the protein of interest and further interact with the amino acids GLN167, PHE168, ASN250, etc., through hydrogen bonding or hydrophobic interactions (figures 1 and 2). Detailed information for docking experiment can be found in the electronic supplementary material. In particular, the common interactions for these three compounds were the hydrogen bonding with the amino acid ASN250 of the protein, which is shown to be closely relevant to antagonist interaction [28,29]. Furthermore, the docking results showed that the compound cpd**11** with the highest *K*_i_ value has the highest predicted affinity (−9.08 kcal mol^−1^, table 2).

**Table 2.** The predicted free energy and affinity compared with experimental values.

entry	experimental *K*_i_ (hA_3_AR, nM)	predicted free energy (kcal mol^−1^)	predicted affinity (nM)
11	111 ± 30	−9.08	222.18
13	177 ± 43	−8.73	398.82
9	679 ± 149	−8.38	719.10

Figure 2 depicts the binding mode of the compound cpd**11** in the ligand-binding pocket of the protein in details. Cpd**11** can be well docked into the binding site of interest. In the most potent molecule cpd**11**, the adenosine core contributed strongly to the binding affinity, which also demonstrates the rationality of the core as a key scaffold for the further chemical modifications. The hydroxyl oxygen of furan ring and the 6-postion *N* atom of adenyl group interact with the ASN250, GLN167, PHE168 and VAL178 through hydrogen bonding. On the other hand, the high affinity also requires the presence of the 2-*O*-alkyl-substituted groups for producing the strong hydrophobic interactions.

From these results, we have illustrated the interactions of our newly synthesized 2-*O*-alkyl-substituted adenosine analogues with a ligand-binding site of hA_3_AR from the molecular modelling point of view. The results also showed the different roles of the substitutions which are strongly linked to the increased or decreased affinity. The exploration for these interactions can provide us the guidance for the future chemical modifications.

## 3. Conclusion

In this work, we designed and synthesized a series of 2-*O*-alkyl-substituted adenosine analogues with indole moiety. The 2-substituents **11** was the most potent among the series, and it was confirmed to be a modulator in a functional assay measuring its capacity to bind receptors in CHO cells expressing the hA_3_A receptor. We found that 2-substituents **11** were critical structural determinant for A_3_AR ligands (*K*_i_ = 111 nM). The promising compound can be considered a valuable seed for the design and development of new and even more selective and potent compounds. The molecular modelling studies have also been performed to investigate the binding mode of the potential compound in the ligand-binding pocket of human A_3_ receptor. Here, this study provides useful foundations for the attainment of a detailed pharmacological and physiological characterization of the adenosine A_3_ receptor.

## Supplementary Material

Experimental synthetic and biological procedures

## Data accessibility

The detailed experimental synthetic procedures and spectra of the final compounds are provided in the electronic supplementary material.
