# Supplementary material for: Synthesis of adenosine analogues with indole moiety as human adenosine A3 receptor ligands
Source: R Soc Open Sci. 2018 Feb 7;5(2):171596. doi: 10.1098/rsos.171596 (PMC5830761; doi:10.1098/rsos.171596)
Supplement: Experimental synthetic and biological procedures [file rsos171596supp1.doc]

**Synthesis and biological evaluation of** **adenosine analogues with indole moiety as human adenosine A_3_ receptor ligands**

*Yan Xia, ^a,b,c,†^ Xiliang Zheng, ^a,†^ Erkang Wang, ^a^ Dongfeng Li, ^b,*^ Ruibin Hou ^b, c,*^ and Jin Wang ^a,d,*^*

^a^State Key Laboratory of Electroanalytical Chemistry, Changchun Institute of Applied Chemistry Chinese Academy of Sciences, Renmen Street, Changchun, 130022, China.

^b^College of Chemistry and Life Science, Changchun University of Technology, Yanan Street No. 2055, Changchun, 130012, China.

^c^Advanced Institute of Materials Science, Changchun University of Technology, Yanan Street No. 2055, Changchun, 130012, China.

^d^Department of Chemistry and Physics, State University of New York at Stony Brook, New York, USA.

**Supporting Information**

**Supporting Information**

**Experimental Procedures and Characterization Data**

**General Experimental**

All the commercial chemicals were of reagent grade and were used without further purification. Solvent were dried with standard procedures. All reactions were carried out under an atmosphere of dried argon, in flame-dried glassware. Proton nuclear magnetic resonance (1H NMR) spectra were determined on a Varian (300 MHz) spectrometer. Chemical shifts are provided in parts per million (ppm) downfield from tetramethylsilane (internal standard) with coupling constants in hertz (Hz). Multiplicity is indicated by the following abbreviations: singlet (s), doublet (d), doublet of doublet (dd), triplet (t), quartet (q), multiplet (m), broad (b). Mass spectra were recorded on a HRMS (EI-MS) was obtained on a JMS-700 (Jeol, Japan). Products from all reactions were purified by flash column chromatography using silica gel 60 (230-400 mesh Kieselgel 60) or by preparative thin layer chromatography using glass-backed silica gel plates (1mm thickness) unless otherwise indicated. Additionally, thin-layer chromatography on 0.25 mm silica plates (E. Merck, silica gel 60 F254) was used to monitor reactions. The chromatograms were visualized using ultraviolet illumination, exposure to iodine vapors, dipping in PMA or Hanessian’s solution. The purity of the final products was checked by reversed phase high-pressure liquid chromatography (RP-HPLC), which was performed on Dionex Corp. HPLC system equipped with a UV detector set at 254 nm. The mobile phases used were A: H_2_O containing 0.05 % TFA, and B: CH_3_CN. The HPLC employed an YMC Hydrosphere C18 (HS-302) column (5μm particle size, 12 nm pore size), 4.6 mm dia. x 150 mm with a flow rate of 1.0 mL/min. Compound purity was assessed using one of the following systems, System A: gradient 20 % B to 100 % B in 30 min; System B: gradient 25 % B to 100 % B in 30 min.

**Scheme 1.** Synthesis of compounds **9-14**.

**Scheme 2.** Synthesis of intermediates **19a-b**.

**Scheme 3.** Synthesis of intermediates **25a-b**.

**Scheme 4.** Synthesis of intermediates **32a-b**.

**Experimental Procedures**

**Ethyl 2-(6-bromo-1H-indol-3-yl)-2-oxoacetate (16)**

To a solution of 6-bromoindole **15** (400 mg, 2.04 mmol) in 20 mL of Et_2_O was added oxalyl chloride (0.45 mL, 5.12 mmol) at 0 ˚C and stirred at room temperature for 10 h. After evaporation, 20 mL of ethanol was added to the solids and continued to stir at room temperature overnight. Evaporation and washed with water, extracted with EtOAc and dried over MgSO_4_, filtered and concentrated in vacuo. The resulting solid was dissolved in EtOAc and precipitated from hexane, filtered and dried to obtain crude product 524 mg without further purification. ^1^H NMR (DMSO-*d_6_*, 300 MHz) *δ* 12.45 (b, 1H), 8.46 (s, 1H), 8.09 (d, *J* = 8.4 Hz, 1H), 7.75 (d, *J* = 1.2 Hz, 1H), 7.42 (dd, *J* = 1.8 Hz, 8.4 Hz, 1H), 4.36 (q, *J* = 7.2 Hz, 2H), 1.34 (t, *J* = 6.9 Hz, 3H).

**General procedure for free amine protection 17a and 17b**

A mixture of **16** (1.0 equiv) and K_2_CO_3_ (5.0 equiv) were suspended in anhydrous acetone and halide (2.0 equiv) was added at room temperature after stirring vigorously for 30 min. The reaction mixture was heated to reflux until starting material disappeared monitored by TLC. The mixture was evaporated and washed with water, extracted with MC and dried over anhydrous MgSO_4_, filtered and concentrated in vacuo. The crude product was purified by column chromatography on silica gel afforded **17a** and **17b**.

**Ethyl 2-(6-bromo-1-methyl-1H-indol-3-yl)-2-oxoacetate (17a)**

Obtained according to general procedure as a pale yellow solid (420 mg, yield 82 %). ^1^H NMR (CDCl_3_, 300 MHz) *δ* 8.32 (s, 1H), 8.29 (d, *J* = 8.1 Hz, 1H), 7.53 (d, *J* = 1.5 Hz, 1H), 7.45 (dd, *J* = 1.5 Hz, 9.0 Hz, 1H), 4.41 (q, *J* = 6.9 Hz, 2H), 3.85 (s, 3H), 1.44 (t, *J* = 6.9 Hz, 3H); MS (EI) m/z 309 (M^+^).

**Ethyl 2-(1-benzyl-6-bromo-1H-indol-3-yl)-2-oxoacetate (17b)**

Obtained according to general procedure as a pale yellow solid (480 mg, yield 92 %). ^1^H NMR (CDCl_3_, 300 MHz) *δ* 8.39 (s, 1H), 8.32 (d, *J* = 8.4 Hz, 1H), 7.47 (s, 1H), 7.44 (dd, *J* = 1.2 Hz, 7.8 Hz, 1H), 7.35-7.37 (m, 3H), 7.16-7.19 (m, 2H), 5.34 (s, 2H), 4.39 (q, *J* = 6.6 Hz, 2H), 1.41 (t, *J* = 6.6 Hz, 3H); MS (EI) m/z 385 (M^+^).

**General method for reduction 18a and 18b**

The solution of **17a** or **17b** (1.0 equiv) in 15 mL of THF was treated with 2.0 M of BH_3_-SMe_2_ in THF (4.0 equiv) and refluxed for 4 h. The solution was cooled to room temperature and poured into ice-water, extracted with Et_2_O and the organic layers were washed with aqueous NaHCO_3_. Dried over MgSO_4_ and concentrated, purified by column chromatography on silica gel to form **18a** and **18b**.

**2-(6-Bromo-1-methyl-1H-indol-3-yl)ethanol (18a)**

Compound **18a** was prepared from **17a** afforded as a white solid (230 mg, yield 70 %). ^1^H NMR (CDCl_3_, 300 MHz) *δ* 7.46 (d, *J* = 1.8 Hz, 1H), 7.45 (d, *J* = 8.1 Hz, 1H), 7.21 (dd, *J* = 1.8 Hz, 8.4 Hz, 1H), 6.91 (s, 1H), 3.87 (t, *J* = 6.0 Hz, 2H), 3.72 (s, 3H), 2.98 (t, *J* = 6.0 Hz, 2H); MS (EI) m/z 253 (M^+^).

**2-(1-Benzyl-6-bromo-1H-indol-3-yl)ethanol (18b)**

Product **18b** obtained from **17b** as a white solid (182 mg, yield 51 %). ^1^H NMR (CDCl_3_, 300 MHz) *δ* 7.48 (d, *J* = 8.4 Hz, 1H), 7.43 (d, *J* = 0.9 Hz, 1H), 7.28-7.35 (m, 3H), 7.22 (dd, *J* = 1.8 Hz, 8.4 Hz, 1H), 7.09-7.12 (m, 2H), 6.98 (s, 1H), 5.23 (s, 2H), 3.88 (q, *J* = 6.0 Hz, 2H), 3.00 (t, *J* = 6.0 Hz, 2H); MS (EI) m/z 329 (M^+^).

**General iodination procedure for synthesis of 19a and 19b**

Imidazole (1.5 equiv), triphenylphosphine (1.5 equiv) and iodine (2.0 equiv) were added sequentially to a rapidly stirring solution of **18a** or **18b** (1.0 equiv) in 10 mL of benzene at room temperature. The reaction solution was stirred overnight and quenched by a mixture solution of NaHCO_3_ and Na_2_S_2_O_3_. The aqueous mixture was extracted there times with ether and combined organic layers were dried over MgSO_4_ and concentrated under reduced pressure. The crude material was purified by silica gel column chromatography to give the iodide as a pale yellow solid.

**6-Bromo-3-(2-iodoethyl)-1-methyl-1H-indole (19a)**

The yield was 73 %. ^1^H NMR (CDCl_3_, 300 MHz) *δ* 7.46 (d, *J* = 1.8 Hz, 1H), 7.42 (d, *J* = 8.1 Hz, 1H), 7.22 (dd, *J* = 1.8 Hz, 8.4 Hz, 1H), 6.91 (s, 1H), 3.72 (s, 3H), 3.36-3.41 (m, 2H), 3.26-3.32 (m, 2H); MS (EI) m/z 363 (M^+^).

**1-Benzyl-6-bromo-3-(2-iodoethyl)-1H-indole (19b)**

The yield was 54 %. ^1^H NMR (CDCl_3_, 300 MHz) *δ* 7.44 (d, *J* = 8.1 Hz, 1H), 7.42 (d, *J* = 2.1 Hz, 1H), 7.28-7.35 (m, 3H), 7.22 (dd, *J* = 1.5 Hz, 8.1 Hz, 1H), 7.08-7.11 (m, 2H), 6.98 (s, 1H), 5.24 (s, 2H), 3.38-3.51 (m, 2H), 3.27-3.33 (m, 2H); MS (EI) m/z 439 (M^+^).

**Methyl 3-(1H-indol-3-yl)propanoate (21)**

The 3-indolepropionic acid **20** (3 g, 0.02 mol) was dissolved in 20 mL of DMF and KHCO_3_ (3.30 g, 0.03 mol) was added at room temperature. The resulting mixture was stirred for 30 min and CH_3_I (2.0 mL, 0.03 mol) was added and heated at 60 ˚C until starting material disappeared. Cooled to room temperature and washed with water, extracted with EA and dried over MgSO_4_, filtered and concentrated to dryness. The resulting solid was purified by silica gel column chromatography to give white solid **21** (2.64 g, yield 82 %). ^1^H NMR (CDCl_3_, 300 MHz) *δ* 7.96 (b, 1H), 7.61 (d, *J* = 8.1 Hz, 1H), 7.36 (d, *J* = 8.1 Hz, 1H), 7.20 (t, *J* = 7.2 Hz, 1H), 7.13 (t, *J* = 7.2 Hz, 1H), 7.02 (d, *J* = 2.1 Hz, 1H), 3.68 (s, 3H), 3.12 (t, *J* = 7.5 Hz, 2H), 2.74 (t, *J* = 7.5 Hz, 2H); MS (EI) m/z 203 (M^+^).

**General protection procedure for the synthesis of 22a and 22b**

To a solution of the **21** (1.0 equiv) in DMF and 60 % NaH (3.0 equiv) was added. The resulting mixture was stirred for 1 h and halide was added. The reaction mixture was heated at 80 ˚C for 4 h. Cooled to room temperature and quenched with ice-water, extracted with EA and dried over MgSO_4_. The filtrate was evaporated in vacuo and the residue was purified by column chromatography to yield white solid **22a** and **22b**.

**Methyl 3-(1-methyl-1H-indol-3-yl)propanoate (22a)**

According to general procedure gave **22a** 410 mg, yield was 77 %. ^1^H NMR (CDCl_3_, 300 MHz) *δ* 7.59 (d, *J* = 7.8 Hz, 1H), 7.29 (d, *J* = 8.1 Hz, 1H), 7.22 (t, *J* = 7.0 Hz, 1H), 7.11 (t, *J* = 7.2 Hz, 1H), 6.87 (s, 1H), 3.74 (s, 3H), 3.68 (s, 3H), 3.10 (t, *J* = 7.8 Hz, 2H), 2.71 (t, *J* = 7.2 Hz, 2H); MS (EI) m/z 217 (M^+^).

**Methyl 3-(1-benzyl-1H-indol-3-yl)propanoate (22b)**

According to general procedure gave **22b** 570 mg, yield was 74 %. ^1^H NMR (CDCl_3_, 300 MHz) *δ* 7.62 (d, *J* = 7.2 Hz, 1H), 7.25-7.33 (m, 3H), 7.19 (d, *J* = 7.2 Hz, 1H), 7.15 (dd, *J* = 1.5 Hz, 3.6 Hz, 1H), 7.09-7.12 (m, 3H), 6.94 (s, 1H), 5.27 (s, 2H), 3.67 (s, 3H), 3.12 (t, *J* = 7.5 Hz, 2H), 2.73 (t, *J* = 8.1 Hz, 2H); MS (EI) m/z 293 (M^+^).

**General procedure for reduction of ester to afford alcohol 23a, 23b, 30a and 30b**

The compound **22a-b** or **29a-b** (1.0 equiv) was dissolved in 10 mL of THF and solution of LiAlH_4_ in THF (1.5 equiv) was added at 0 ˚C. The reaction solution was stirred at room temperature for 2 h and quenched by 10 % HCl and ice-water. The mixture was extracted there times with ether. Dried over anhydrous MgSO_4_ and filtered, concentrated and the residue was purified by column chromatography obtained white solid **23a-b** and **30a-b**.

**3-(1-Methyl-1H-indol-3-yl)propan-1-ol (23a)**

Obtained from **22a** as a colorless oil (510 mg, yield 87 %). ^1^H NMR (CDCl_3_, 300 MHz) *δ* 7.61 (d, *J* = 7.8 Hz, 1H), 7.30 (d, *J* = 8.1 Hz, 1H), 7.23 (dd, *J* = 1.2 Hz, 6.9 Hz, 1H), 7.10 (dt, *J* = 1.2 Hz, 8.1 Hz, 1H), 6.86 (s, 1H), 3.75 (s, 3H), 3.74 (q, *J* = 6.3 Hz, 2H), 2.86 (t, *J* = 7.2 Hz, 2H), 1.94-2.03 (m, 2H), 1.30 (t, *J* = 4.2 Hz, 1H); MS (EI) m/z 189 (M^+^).

**3-(1-Benzyl-1H-indol-3-yl)propan-1-ol (23b)**

Obtained from **22b** as a white solid (500 mg, yield 97 %. ^1^H NMR (CDCl_3_, 300 MHz) *δ* 7.62 (d, *J* = 7.8 Hz, 1H), 7.24-7.32 (m, 3H), 7.16 (dd, *J* = 1.5 Hz, 8.1 Hz, 1H), 7.08-7.14 (m, 3H), 6.93 (s, 1H), 5.28 (s, 2H), 3.73 (q, *J* = 6.0 Hz, 2H), 2.87 (t, *J* = 7.2 Hz, 2H), 1.95-2.05 (m, 2H), 3.60 (t, *J* = 4.5 Hz, 1H); MS (EI) m/z 265 (M^+^).

**3-(6-Bromo-1-methyl-1H-indol-3-yl)propan-1-ol (30a)**

Obtained from **29a** as a colorless oil (276 mg, yield 100 %). ^1^H NMR (CDCl_3_, 300 MHz) *δ* 7.45 (d, *J* = 6.6 Hz, 1H), 7.43 (s, 1H), 7.19 (dd, *J* = 1.5 Hz, 8.1 Hz, 1H), 6.82 (s, 1H), 3.69-3.74 (m, 2H), 3.70 (s, 3H), 2.81 (t, *J* = 7.2 Hz, 2H), 1.90-1.99 (m, 2H); MS (EI) m/z 267 (M^+^).

**3-(1-Benzyl-6-bromo-1H-indol-3-yl)propan-1-ol (30b)**

Obtained from **29b** as a colorless oil (256 mg, yield 97 %). ^1^H NMR (CDCl_3_, 300 MHz) *δ* 7.48 (d, *J* = 9.0 Hz, 1H), 7.41 (d, *J* = 1.8 Hz, 1H), 7.26-7.35 (m, 3H), 7.21 (dd, *J* = 1.5 Hz, 8.7 Hz, 1H), 7.08-7.12 (m, 2H), 6.89 (s, 1H), 5.21 (s, 2H), 3.71 (t, *J* = 6.0 Hz, 2H), 2.83 (t, *J* = 7.8 Hz, 2H), 1.91-2.01 (m, 2H) ; MS (EI) m/z 343 (M^+^).

**General tosylation procedure for preparation of 24a-b and 31a-b**

The compound **23a-b** or **30a-b** (1.0 equiv) was dissolved in the 12 mL of DCM and DMAP (0.1 equiv), Et_3_N (3.0 equiv) and TsCl (1.5 equiv) were added at 0 ˚C. The reaction solution was stirred at room temperature overnight and evaporated in vacuo. The residue was purified by column chromatography to give compounds **24a-b** and **31a-b**.

**3-(1-Methyl-1H-indol-3-yl)propyl 4-methylbenzenesulfonate (24a)**

Obtained as a colorless oil from corresponding alcohol **23a** (520 mg, yield 56 %). ^1^H NMR (CDCl_3_, 300 MHz) *δ* 7.79 (d, *J* = 8.4 Hz, 2H), 7.49 (d, *J* = 7.8 Hz, 1H), 7.31 (d, *J* = 8.4 Hz, 2H), 7.19-7.29 (m, 2H), 7.09 (dt, *J* = 0.9 Hz, 6.9 Hz, 1H), 6.70 (s,1H), 4.08 (t, *J* = 5.7 Hz, 2H), 3.69 (s, 3H), 2.80 (t, *J* = 7.2 Hz, 2H), 2.44 (s, 3H), 2.02 (m, 2H); MS (EI) m/z 343 (M^+^).

**3-(1-Benzyl-1H-indol-3-yl)propyl 4-methylbenzenesulfonate (24b)**

Obtained as a colorless oil from corresponding alcohol **23b** (301 mg, yield 95 %). ^1^H NMR (CDCl_3_, 300 MHz) *δ* 7.77 (d, *J* = 8.1 Hz, 2H), 7.50 (d, *J* = 8.1 Hz, 1H), 7.25-7.32 (m, 5H), 7.22 (s, 1H), 7.16 (dt, *J* = 1.5 Hz, 7.5 Hz, 1H), 7.05-7.10 (m, 3H), 6.79 (s, 1H), 5.23 (s, 2H), 4.08 (t, *J* = 6.6 Hz, 2H), 2.81 (t, *J* = 6.6 Hz, 2H), 2.41 (s, 3H), 2.04 (m, 2H); MS (EI) m/z 419 (M^+^).

**3-(6-Bromo-1-methyl-1H-indol-3-yl)propyl 4-methylbenzenesulfonate (31a)**

Obtained as a colorless oil from corresponding alcohol **30a** (329 mg, yield 76 %). ^1^H NMR (CDCl_3_, 300 MHz) *δ* 7.78 (d, *J* = 8.4 Hz, 2H), 7.41 (d, *J* = 1.5 Hz, 1H), 7.32 (d, *J* = 8.1 Hz, 2H), 7.31 (s, 1H), 7.15 (dd, *J* = 1.5 Hz, 8.1 Hz, 1H), 6.70 (s, 1H), 4.06 (t, *J* = 6.0 Hz, 2H), 3.66 (s, 3H), 2.76 (t, *J* = 7.2 Hz, 2H), 2.45 (s, 3H), 1.99 (m, 2H); MS (EI) m/z 421 (M^+^).

**3-(1-Benzyl-6-bromo-1H-indol-3-yl)propyl 4-methylbenzenesulfonate (31b)**

Obtained as a colorless oil from corresponding alcohol **30b** (312 mg, yield 86 %). ^1^H NMR (CDCl_3_, 300 MHz) *δ* 7.76 (d, *J* = 8.4 Hz, 2H), 7.38 (d, *J* = 1.8 Hz, 1H), 7.34 (d, *J* = 8.7 Hz, 2H), 7.26-7.32 (m, 4H), 7.16 (dd, *J* = 1.8 Hz, 8.4 Hz, 1H), 7.04-7.07 (m, 2H), 6.77 (s, 1H), 5.18 (s, 2H), 4.06 (t, *J* = 6.0 Hz, 2H), 2.77 (t, *J* = 7.2 Hz, 2H), 2.42 (s, 3H), 2.01 (m, 2H); MS (EI) m/z 497 (M^+^).

**General iodination procedure for synthesis of 25a-b and 32a-b**

The mixture of **24a-b** or **31a-b** (1.0 equiv) and KI (3.0 equiv) were suspended in anhydrous acetone and refluxed overnight. The mixture was evaporated under reduced pressure and the resulting material was purified by column chromatography formed colorless oil **25a-b** and **32a-b**.

**3-(3-Iodopropyl)-1-methyl-1H-indole (25a)**

Formed according to general procedure (242 mg, yield 50 %). ^1^H NMR (CDCl_3_, 300 MHz) *δ* 7.62 (d, *J* = 7.5 Hz, 1H), 7.32 (d, *J* = 7.5 Hz, 1H), 7.26 (dt, *J* = 1.2 Hz, 7.2 Hz, 1H), 7.13 (dt, *J* = 1.8 Hz, 8.1 Hz, 1H), 6.91 (s, 1H), 3.76 (s, 3H), 3.24 (t, *J* = 7.2 Hz, 2H), 2.90 (t, *J* = 7.2 Hz, 2H), 2.21 (m, 2H); MS (EI) m/z 299 (M^+^).

**1-Benzyl-3-(3-iodopropyl)-1H-indole (25b)**

Formed according to general procedure (110 mg, yield 41 %). ^1^H NMR (CDCl_3_, 300 MHz) *δ* 7.63 (d, *J* = 8.4 Hz, 1H), 7.26-7.34 (m, 4H), 7.20 (dd, *J* = 0.9 Hz, 6.6 Hz, 1H), 7.16 (dd, *J* = 1.2 Hz, 5.1 Hz, 1H), 7.10-7.13 (m, 2H), 6.98 (s, 1H), 5.29 (s, 2H), 3.23 (t, *J* = 7.2 Hz, 2H), 2.90 (t, *J* = 7.2 Hz, 2H), 2.21 (m, 2H); MS (EI) m/z 375 (M^+^).

**6-Bromo-3-(3-iodopropyl)-1-methyl-1H-indole (32a)**

Formed according to general procedure (215 mg, yield 73 %). ^1^H NMR (CDCl_3_, 300 MHz) *δ* 7.45 (d, *J* = 1.5 Hz, 1H), 7.42 (d, *J* = 8.1 Hz, 1H), 7.20 (dd, *J* = 1.8 Hz, 8.4 Hz, 1H), 6.86 (s, 1H), 3.71 (s, 3H), 3.20 (t, *J* = 6.6 Hz, 2H), 2.84 (t, *J* = 7.2 Hz, 2H), 2.17 (m, 2H); MS (EI) m/z 377 (M^+^).

**1-Benzyl-6-bromo-3-(3-iodopropyl)-1H-indole (32b)**

Formed according to general procedure (252 mg, yield 89 %). ^1^H NMR (CDCl_3_, 300 MHz) *δ* 7.47 (d, *J* = 8.1 Hz, 1H), 7.42 (d, *J* = 1.5 Hz, 1H), 7.28-7.33 (m, 3H), 7.22 (dd, *J* = 1.8 Hz, 8.4 Hz, 1H), 7.08-7.11 (m, 2H), 6.94 (s, 1H), 5.23 (s, 2H), 3.20 (t, *J* = 6.6 Hz, 2H), 2.86 (t, *J* = 6.6 Hz, 2H), 2.17 (m, 2H); MS (EI) m/z 453 (M^+^).

**General procedure for alkylation of 6-bromoindole-3-carboxaldehyde**

The 6-bromoindole-3-carboxaldehyde **26** (1.0 equiv) was dissolved in the 15 mL of anhydrous acetone and K_2_CO_3_ (5.0 equiv) was added. The mixture was stirred at room temperature for 30 min and halide (1.5 equiv) was added. The reaction mixture was refluxed at 60 ˚C until starting material disappeared and evaporated under reduced pressure. The residue was washed with water and extracted with MC and dried over MgSO_4_. The filtrate was concentrated and purified by column chromatography on silica gel to yield white solid **27a** or **27b**.

**6-Bromo-1-methyl-1H-indole-3-carbaldehyde (27a)**

The yield was 96 %. ^1^H NMR (CDCl_3_, 300 MHz) *δ* 9.97 (s, 1H), 8.16 (d, *J* = 8.4 Hz, 1H), 7.65 (s, 1H), 7.53 (d, *J* = 1.5 Hz, 1H), 7.43 (dd, *J* = 1.8 Hz, 8.4 Hz, 1H), 3.84 (s, 3H); MS (EI) m/z 237 (M^+^).

**1-Benzyl-6-bromo-1H-indole-3-carbaldehyde (27b)**

The yield was 87 %. ^1^H NMR (CDCl_3_, 300 MHz) *δ* 9.98 (s, 1H), 8.20 (d, *J* = 8.7 Hz, 1H), 7.68 (s, 1H), 7.50 (d, *J* = 1.2 Hz, 1H), 7.43 (dd, *J* = 1.8 Hz, 8.4 Hz, 1H), 7.35-7.39 (m, 3H), 7.17-7.19 (m, 2H), 5.32 (s, 2H); MS (EI) m/z 313 (M^+^).

**General Wittig reaction procedure for synthesis of 28a-b**

The compound **27a-b** (1.0 equiv) was dissolved in the 15 mL of DCM and methyl(triphenylphosphoranylidene)acetate (2.0 equiv) was added. The resulting solution was refluxed for 16 h and evaporated under reduced pressure. The crude product was purified by column chromatography on silica gel gave **28a-b**.

**(E)-methyl 3-(6-bromo-1-methyl-1H-indol-3-yl)acrylate (28a)**

According to general procedure to obtain white solid (336 mg, yield 68 %). ^1^H NMR (CDCl_3_, 300 MHz) *δ* 7.84 (d, *J* = 15.9 Hz, 1H), 7.74 (d, *J* = 8.7 Hz, 1H), 7.50 (d, *J* = 1.8 Hz, 1H), 7.35 (dd, *J* = 1.8 Hz, 9.0 Hz, 1H), 7.31 (s, 1H), 6.36 (d, *J* = 15.9 Hz, 1H), 3.80 (s, 3H), 3.78 (s, 3H); MS (EI) m/z 293 (M^+^).

**(E)-methyl 3-(1-benzyl-6-bromo-1H-indol-3-yl)acrylate (28b)**

According to general procedure to obtain white solid (340 mg, yield 72 %). ^1^H NMR (CDCl_3_, 300 MHz) *δ* 7.84 (d, *J* = 16.5 Hz, 1H), 7.76 (d, *J* = 8.7 Hz, 1H), 7.47 (s, 1H), 7.26-7.36 (m, 5H), 7.12-7.14 (m, 2H), 6.38 (d, *J* = 15.9 Hz, 1H), 5.27 (s, 2H), 3.80 (s, 3H); MS (EI) m/z 369 (M^+^).

**General procedure of the reduction double bond for synthesis of 29a-b**

The **28a-b** (1.0 equiv) was dissolved in the 10 mL of ethanol and BiCl_3_ (3.0 equiv) was added at 0 ˚C and portionwise NaBH_4_. The reaction mixture was stirred at 0 ˚C overnight and filtered through a Celite pad. The filtrate was concentrated and washed with water. The mixture was extracted with EA. The organic layers were dried over MgSO_4_ and filtered. The filtrate was evaporated under reduced pressure and the residue was purified by column chromatography yielded white solid **29a-b**.

**Methyl 3-(6-bromo-1-methyl-1H-indol-3-yl)propanoate (29a)**

The yield was 71 %. ^1^H NMR (CDCl_3_, 300 MHz) *δ* 7.43 (d, *J* = 1.5 Hz, 1H), 7.42 (d, *J* = 8.1 Hz, 1H), 7.20 (dd, *J* = 1.5 Hz, 8.7 Hz, 1H), 6.83 (s, 1H), 3.69 (s, 3H), 3.67 (s, 3H), 3.06 (t, *J* = 7.2 Hz, 2H), 2.68 (t, *J* = 7.5 Hz, 2H); MS (EI) m/z 295 (M^+^).

**Methyl 3-(1-benzyl-6-bromo-1H-indol-3-yl)propanoate (29b)**

The yield was 80 %. ^1^H NMR (CDCl_3_, 300 MHz) *δ* 7.47 (d, *J* = 9.0 Hz, 1H), 7.41 (d, *J* = 1.5 Hz, 1H), 7.26-7.32 (m, 3H), 7.22 (dd, *J* = 1.8 Hz, 8.4 Hz, 1H), 7.06-7.09 (m, 2H), 6.90 (s, 1H), 5.21 (s, 2H), 3.66 (s, 3H), 3.08 (t, *J* = 7.5 Hz, 2H), 2.70 (t, *J* = 7.8 Hz, 2H); MS (EI) m/z 371 (M^+^).

**General synthetic procedure for 2-substituted adenosine derivatives**

To a solution of 6-chloro-2-hydroxy-9-(2,3,5-tri-*O*-acetyl-β-D-ribofuranosyl) purine in DMF were added iodide (1.8 equiv) and Cs_2_CO_3_ (2.7 equiv) at room temperature, and the reaction mixture was stirred overnight. After dilution with ethyl acetate, the solution was washed with water twice, dried over MgSO_4_, and filtered. The filtrate was evaporated to give a crude oil that was purified by column chromatography on silica gel. Elution with a mixture of EA and hexane (2:3) gave the 2-substitued 2',3',5'-triacetyl-6-chloroadenosine derivative.

A solution of 2-substitued 2',3',5'-triacetyl-6-chloroadenosine derivative in mixture of methanol and ammonia hydroxide was stirred in sealed tube overnight at 80 ˚C. The solvent was evaporated to give oil, which was subjected to preparative TLC developed with a mixture of MC and methanol (9:1) to give the 2-substituted adenosine derivative.

**6-Chloro-2-[3"-(6"-bromo-1"-methyl-indolyl)ethoxy]-2',3',5'-triacetyladenosine (3)**

The yield was 37 %. ^1^H NMR (CDCl_3_, 300 MHz) *δ* 8.01 (s, 1H), 7.49 (d, *J* = 9.0 Hz, 1H), 7.38 (d, *J* = 1.8 Hz, 1H), 7.16 (dd, *J* = 1.8 Hz, 9.0 Hz, 1H), 6.96 (s, 1H), 6.06 (d, *J* = 5.1 Hz, 1H), 5.83 (t, *J* = 4.8 Hz, 1H), 5.56 (t, *J* = 5.1 Hz, 1H), 4.60 (m, 2H), 4.24-4.40 (m, 3H), 3.66 (s, 3H), 3.20 (t, *J* = 7.2 Hz, 2H), 2.07 (s, 3H), 2.01 (s, 3H), 2.00 (s, 3H); MS (EI) m/z 663 (M^+^).

**6-Chloro-2-[3"-(6"-bromo-1"-benzyl-indolyl)ethoxy]-2',3',5'-triacetyladenosine (4)**

The yield was 30 %. ^1^H NMR (CDCl_3_, 300 MHz) δ 8.01 (s, 1H), 7.53 (d, J = 9.0 Hz, 1H), 7.34 (d, J = 1.8 Hz, 1H), 7.20-7.24 (m, 3H), 7.17 (dd, J = 1.8 Hz, 8.4 Hz, 1H), 7.01-7.04 (m, 3H), 6.05 (d, J = 4.8 Hz, 1H), 5.82 (t, J = 4.8 Hz, 1H), 5.56 (t, J = 5.4 Hz, 1H), 5.18 (s, 2H), 4.62 (m, 2H), 4.22-4.39 (m, 3H), 3.22 (t, J = 7.2 Hz, 2H), 2.05 (s, 3H), 2.01 (s, 3H), 1.99 (s, 3H); MS (EI) m/z 739 (M^+^).

**6-Chloro-2-[3"-(1"-methyl-indolyl)propoxy]-2',3',5'-triacetyladenosine (5)**

The yield was 28 %. ^1^H NMR (CDCl_3_, 300 MHz) *δ* 8.07 (s, 1H), 7.61 (d, *J* = 7.5 Hz, 1H), 7.29 (d, *J* = 8.1 Hz, 1H), 7.21 (dt, *J* = 1.2 Hz, 8.1 Hz, 1H), 7.08 (dt, *J* = 1.2 Hz, 8.4 Hz, 1H), 6.90 (s, 1H), 6.14 (d, *J* = 4.8 Hz, 1H), 5.89 (t, *J* = 5.7 Hz, 1H), 5.62 (t, *J* = 4.8 Hz, 1H), 4.50 (m, 2H), 4.29-4.46 (m, 3H), 3.75 (s, 3H), 2.98 (t, *J* = 6.9 Hz, 2H), 2.25 (m, 2H), 2.14 (s, 3H), 2.09 (s, 3H), 2.08 (s, 3H); MS (EI) m/z 599 (M^+^).

**6-Chloro-2-[3"-(1"-benzyl-indolyl)propoxy]-2',3',5'-triacetyladenosine (6)**

The yield was 41 %. ^1^H NMR (CDCl_3_, 300 MHz) *δ* 8.08 (s, 1H), 7.63 (d, *J* = 7.5 Hz, 1H), 7.22-7.30 (m, 4H), 7.06-7.18 (m, 4H), 6.98 (s, 1H), 6.15 (d, *J* = 4.8 Hz, 1H), 5.89 (t, *J* = 5.7 Hz, 1H), 5.62 (t, *J* = 5.1 Hz, 1H), 5.28 (s, 2H), 4.51 (m, 2H), 4.28-4.46 (m, 3H), 3.00 (t, *J* = 7.2 Hz, 2H), 2.26 (m, 2H), 2.14 (s, 3H), 2.09 (s, 3H), 2.07 (s, 3H); MS (EI) m/z 675 (M^+^).

**6-Chloro-2-[3"-(6"-bromo-1"-methyl-indolyl)propoxy]-2',3',5'-triacetyladenosine (7)**

The yield was 31 %. ^1^H NMR (CDCl_3_, 300 MHz) *δ* 8.07 (s, 1H), 7.45 (d, *J* = 9.0 Hz, 1H), 7.42 (d, *J* = 1.5 Hz, 1H), 7.17 (dd, *J* = 1.5 Hz, 8.1 Hz, 1H), 6.87 (s, 1H), 6.12 (d, *J* = 4.8 Hz,1H), 5.91 (t, *J* = 5.4 Hz, 1H), 5.62 (t, *J* = 5.4 Hz, 1H), 4.48 (m, 2H), 4.28-4.44 (m, 3H), 3.70 (s, 3H), 2.94 (t, *J* = 7.2 Hz, 2H), 2.22 (m, 2H), 2.14 (s, 3H), 2.08 (s, 3H), 2.07 (s, 3H); MS (EI) m/z 677 (M^+^).

**6-Chloro-2-[3"-(6"-bromo-1"-benzyl-indolyl)propoxy]-2',3',5'-triacetyladenosine (8)**

The yield was 51 %. ^1^H NMR (CDCl_3_, 300 MHz) *δ* 8.07 (s, 1H), 7.48 (d, *J* = 8.7 Hz, 1H), 7.39 (d, *J* = 1.5 Hz, 1H), 7.25-7.29 (m, 3H), 7.18 (dd, *J* = 1.8 Hz, 8.7 Hz, 1H), 7.07-7.11 (m, 2H), 6.94 (s, 1H), 6.13 (d, *J* = 5.1 Hz,1H), 5.90 (t, *J* = 5.7 Hz, 1H), 5.63 (t, *J* = 5.4 Hz, 1H), 5.22 (s, 2H), 4.49 (m, 2H), 4.27-4.44 (m, 3H), 2.95 (t, *J* = 8.7 Hz, 2H), 2.23 (m, 2H), 2.13 (s, 3H), 2.09 (s, 3H), 2.07 (s, 3H); MS (EI) m/z 753 (M^+^).

**2-(1"-Methyl-3"-indolylethoxy)adenosine (9)**

The yield was 28 %. ^1^H NMR (CD_3_OD, 300 MHz) *δ* 8.12 (s, 1H), 7.52 (d, *J* = 8.1 Hz, 1H), 7.50 (d, *J* = 1.8 Hz, 1H), 7.15 (dd, *J* = 1.8 Hz, 8.4 Hz, 1H), 7.11 (s, 1H), 5.90 (d, *J* = 5.4 Hz, 1H), 4.71 (t, *J* = 5.4 Hz, 1H), 4.55 (m, 2H), 4.32 (dd, *J* = 3.9 Hz, 5.7 Hz, 1H), 4.11 (q, *J* = 3.3 Hz, 1H), 3.86 (dd, *J* = 2.7 Hz, 12.3 Hz, 1H), 3.73 (s, 3H), 3.72 (dd, *J* = 3.3 Hz, 12.0 Hz, 1H), 3.17 (t, *J* = 7.2 Hz, 2H); Purity 100 % (as determined by RP-HPLC, method A, *t*_R_ = 7.78 min, method B, *t*_R_ = 9.37 min).

**2-(1"-Benzyl-3"-indolylethoxy)adenosine (10)**

The yield was 28 %. ^1^H NMR (CD_3_OD, 300 MHz) *δ* 8.12 (s, 1H), 7.55 (d, *J* = 8.4 Hz, 1H), 7.42 (d, *J* = 1.5 Hz, 1H), 7.21-7.26 (m, 4H), 7.15 (dd, *J* = 1.5 Hz, 8.1 Hz, 1H), 7.09-7.11 (m, 2H), 5.89 (d, *J* = 5.4 Hz, 1H), 5.30 (s, 2H), 4.70 (t, *J* = 5.4 Hz, 1H), 4.58 (m, 2H), 4.31 (dd, *J* = 3.9 Hz, 5.7 Hz, 1H), 4.10 (q, *J* = 3.3 Hz, 1H), 3.84 (dd, *J* = 3.0 Hz, 12.3 Hz, 1H), 3.72 (dd, *J* = 3.3 Hz, 12.6 Hz, 1H), 3.20 (t, *J* = 6.9 Hz, 2H), 2.89 (t, *J* = 7.2 Hz, 2H), 2.12 (m, 2H); Purity 100 % (as determined by RP-HPLC, method A, *t*_R_ = 11.06 min, method B, *t*_R_ = 12.55 min).

**2-(6"-Bromo-1"-Methyl-3"-indolylpropoxy)adenosine (11)**

The yield was 30 %. ^1^H NMR (CD_3_OD, 300 MHz) *δ* 8.11 (s, 1H), 7.47 (d, *J* = 1.8 Hz, 1H), 7.45 (d, *J* = 8.1 Hz, 1H), 7.08 (dd, *J* = 1.5 Hz, 8.1 Hz, 1H), 6.97 (s, 1H), 5.87 (d, *J* = 6.0 Hz, 1H), 4.71 (t, *J* = 5.1 Hz, 1H), 4.34 (t, *J* = 6.6 Hz, 2H), 4.32 (dd, *J* = 3.3 Hz, 5.1 Hz, 1H), 4.10 (q, *J* = 3.3 Hz, 1H), 3.84 (dd, *J* = 2.7 Hz, 12.0 Hz, 1H), 3.72 (dd, *J* = 4.5 Hz, 11.2 Hz, 1H), 3.70 (s, 3H), 2.89 (t, *J* = 7.2 Hz, 2H), 2.12 (m, 2H); Purity 100 % (as determined by RP-HPLC, method A, *t*_R_ = 5.82 min, method B, *t*_R_ = 7.26 min).

**2-(6"-Bromo-1"-Benzyl-3"-indolylpropoxy)adenosine (12)**

The yield was 30 %. ^1^H NMR (CD_3_OD, 300 MHz) *δ* 8.12 (s, 1H), 7.48 (d, *J* = 8.1 Hz, 1H), 7.42 (d, *J* = 1.8 Hz, 1H), 7.20-7.28 (m, 3H), 7.07-7.11 (m, 4H), 5.88 (d, *J* = 6.0 Hz, 1H), 5.28 (s, 2H), 4.71 (t, *J* = 5.4 Hz, 1H), 4.33 (t, *J* = 6.6 Hz, 2H), 4.31 (dd, *J* = 2.4 Hz, 5.1 Hz, 1H), 4.10 (q, *J* = 3.3 Hz, 1H), 3.84 (dd, *J* = 2.7 Hz, 12.0 Hz, 1H), 3.72 (dd, *J* = 3.3 Hz, 12.0 Hz, 1H), 2.92 (t, *J* = 6.9 Hz, 2H), 2.13 (m, 2H); Purity 100 % (as determined by RP-HPLC, method A, *t*_R_ = 9.62 min, method B, *t*_R_ = 11.17 min).

**2-(6"-Bromo-1"-Methyl-3"-indolylpropoxy)adenosine (13)**

The yield was 28 %. ^1^H NMR (CD_3_OD, 300 MHz) *δ* 8.11 (s, 1H), 7.53 (d, *J* = 7.5 Hz, 1H), 7.28 (d, *J* = 8.1 Hz, 1H), 7.12 (dt, *J* = 0.9 Hz, 8.1 Hz, 1H), 6.99 (dd, *J* = 1.2 Hz, 7.2 Hz, 1H), 6.95 (s, 1H), 5.88 (d, *J* = 5.4 Hz, 1H), 4.71 (t, *J* = 5.7 Hz, 1H), 4.35 (t, *J* = 6.6 Hz, 2H), 4.31 (dd, *J* = 3.3 Hz, 4.8 Hz, 1H), 4.10 (q, *J* = 3.3 Hz, 1H), 3.84 (dd, *J* = 3.0 Hz, 12.3 Hz, 1H), 3.71 (dd, *J* = 3.3 Hz, 12.0 Hz, 1H), 2.92 (t, *J* = 6.9 Hz, 2H), 2.14 (m, 2H); Purity 100 % (as determined by RP-HPLC, method A, *t*_R_ = 8.18 min, method B, *t*_R_ = 9.75 min).

**2-(6"-Bromo-1"-Benzyl-3"-indolylpropoxy)adenosine (14)**

The yield was 24 %. ^1^H NMR (CD_3_OD, 300 MHz) *δ* 8.12 (s, 1H), 7.56 (d, *J* = 7.5 Hz, 1H), 7.17-7.26 (m, 4H), 7.04-7.09 (m, 4H), 6.98 (t, *J* = 6.9 Hz, 1H), 5.88 (d, *J* = 5.4 Hz, 1H), 5.30 (s, 2H), 4.71 (t, *J* = 5.4 Hz, 1H), 4.34 (t, *J* = 6.6 Hz, 2H), 4.32 (dd, *J* = 3.9 Hz, 4.8 Hz, 1H), 4.10 (q, *J* = 3.3 Hz, 1H), 3.84 (dd, *J* = 3.0 Hz, 12.3 Hz, 1H), 3.72 (dd, *J* = 3.3 Hz, 12.9 Hz, 1H), 2.95 (t, *J* = 7.2 Hz, 2H), 2.13-2.18 (m, 2H); Purity 100 % (as determined by RP-HPLC, method A, *t*_R_ = 11.58 min, method B, *t*_R_ = 13.05 min).

**Biological assay procedures**

**Pharmacological methods**

[^125^I]*N*^6^-(4-amino-3-iodobenzyl)-adenosine-5’-*N*-methyluronamide (I-AB-MECA; 2000 Ci/mmol), [^3^H]CCPA (2-chloro-*N*^6^-cyclopentyladenosine, 42.6 Ci/mmol), [^3^H]-CGS21680 (2-[*p*-(2-carboxyethyl)phenylethylamino]-5’-*N*-ethylcarboxamido-adenosine, 47 Ci/mmol), and [^3^H]cyclic AMP (40 Ci/mmol) were from Amersham Pharmacia Biotech (Buckinghamshire, U. K.).

**Cell culture and membrane preparation**

CHO (Chinese hamster ovary) cells expressing the recombinant human ARs^2^ were cultured in DMEM supplemented with 10% fetal bovine serum, 100 units/mL penicillin, 100 *µ*g/mL streptomycin, 2 *µ*mol/mL glutamine, and 800 *µ*g/mL geneticin. Cells were harvested by trypsinization. After homogenization and suspension, cells were centrifuged at 500 *g* for 10 min, and the pellet was resuspended in 50 mM Tris-HCl buffer (pH 8.0) containing 10 mM MgCl_2_, 1 mM EDTA, and 0.1 mg/mL CHAPS. The suspension was homogenized with an electric homogenizer for 10 s and was then recentrifuged at 20000 *g* for 20 min at 4 °C. The resultant pellets were resuspended in buffer in the presence of 3 units/mL adenosine deaminase, and the suspension was stored at -80 °C until the binding experiments. The protein concentration was measured using the Bradford assay.^3^

**Binding assay**

Human A_1_ and A_2A_ receptors: For binding to human A_1_ receptors, [^3^H]CCPA (1 nM) was incubated with membranes (40 *µ*g/tube) from CHO cells stably expressing human A_1_ receptors at 25 °C for 60 min in 50 mM Tris-HCl buffer (pH 7.4; MgCl_2_, 10 mM) in a total assay volume of 200 *µ*L. Nonspecific binding was determined using 10 *µ*M of CPA. For human A_2A_ receptor binding, membranes (20 *µ*g/tube) from HEK-293 cells stably expressing human A_2A_ receptors were incubated with 15 nM [^3^H]CGS21680 at 25 °C for 60 min in 200 *µ*L of 50 mM Tris-HCl, pH 7.4, containing 10 mM MgCl_2_. NECA (10 *µ*M) was used to define nonspecific binding. Reaction was terminated by filtration with GF/B filters.

Human A_3_ receptor: For competitive binding assay, each tube contained 100 *µ*L of membrane suspension (20 *µ*g protein), 50 *µ*L of [^125^I]I-AB-MECA (0.5 nM), and 50 *µ*L of increasing concentrations of the nucleoside derivative in Tris-HCl buffer (50 mM, pH 7.4) containing 10 mM MgCl_2_ and 1 mM EDTA. Nonspecific binding was determined using 10 *µ*M of Cl-IB-MECA in the buffer. The mixtures were incubated at 25 °C for 60 min. Binding reactions were terminated by filtration through Whatman GF/B filters under reduced pressure using a MT-24 cell harvester (Brandell, Gaithersburgh, MD). Filters were washed three times with 9 mL of ice-cold buffer. Radioactivity was determined in a Beckman 5500B *γ*-counter.

**Molecular Docking**

All docking calculations were carried out using the homology model of the hA_3_AR (PDB id: 1OEA),^4^ which was derived from the X-ray structure of bovine rhodopsin (PDB id: 1F88).^5^ This homology structure had been proved to be the start structure which can be used for the molecular docking calculations.^6^ The binding site was identified according to the previous studies.^4^ The residues (L91, T94, Q167, F168, N250, H272, N274) were chosen out to frame the ligand-binding pocket. The structures of all compounds were generated and optimized by openbabel.^7^ For the protein, the side chains and termini protons are assigned using the corresponding protonation states at pH 7.0 as a template. The initial preparation regarding the conformations is determined by the AutoDock Tools. All docking calculations were performed with the AutoDock package to sample the conformation space of the ligands. All compounds are docked by targeting the binding site of hA_3_AR. The Autogrid, with 40×40×40 grid size and value of 0.375 Å spacing centered on the special position in the binding pocket, is also prepared using the AutoDock tools. Dockings are performed using the AutoDock4.2 score function for evaluation and Lamarckian genetic algorithm (LGA) for sampling docked poses. Molecular modeling is carried out based on the following parameters: the population size of 300 and the energy evaluation of 3,000,000 per run with the maximum number of generations of 27,000. The other parameters are set at default values. The number of docking runs was 100. The docking results are evaluated according to the predicted binding affinities. And the subsequent cluster analysis is based on the root mean square deviation (RMSD) value using 2.0 Å as the cutoff.

^1^ Moorman, A. R. Synthesis of 2-aralkoxy adenosines and 2-alkoxyadenosines. WO patent 2003035662, **2003**.

^2^ Z. G. Gao, L. Mamedova, P. Chen, K. A. Jacobson, *Biochem. Pharmacol.* **2004**, *68*, 1985-1993.

^3^ M. M. Bradford, *Anal. Biochem.* **1976**, *72*, 248-254.

^4^ Z. G. Gao, S. K. Kim, T. Biadatti, W. Z. Chen, K. Lee, D. Barak, S. G. Kim, C. R. Johnson, K. A. Jacobson, *J. Med. Chem.*

**2002**, *45*, 4471-4484.

^5^ K. Palczewski, T. Kumasaka, T. Hori, C. A. Behnke, H. Motoshima, B. A. Fox, I. L. Trong, D. C. Teller, T. Okada, R. E.

Stenkamp, M. Yamamoto, M. Miyano, *Science* **2000**, *289*, 739-745.

^6^ [E. M](http://www.ncbi.nlm.nih.gov/pubmed/?term=Priego EM%5BAuthor%5D&cauthor=true&cauthor_uid=18000937). Priego, [M. J](http://www.ncbi.nlm.nih.gov/pubmed/?term=Pérez-Pérez MJ%5BAuthor%5D&cauthor=true&cauthor_uid=18000937). Pérez-Pérez, [J. K](http://www.ncbi.nlm.nih.gov/pubmed/?term=von Frijtag Drabbe Kuenzel JK%5BAuthor%5D&cauthor=true&cauthor_uid=18000937). von Frijtag Drabbe Kuenzel, [H](http://www.ncbi.nlm.nih.gov/pubmed/?term=de Vries H%5BAuthor%5D&cauthor=true&cauthor_uid=18000937). de Vries, [A. P](http://www.ncbi.nlm.nih.gov/pubmed/?term=Ijzerman AP%5BAuthor%5D&cauthor=true&cauthor_uid=18000937). Ijzerman, [M. J](http://www.ncbi.nlm.nih.gov/pubmed/?term=Camarasa MJ%5BAuthor%5D&cauthor=true&cauthor_uid=18000937). Camarasa, [S](http://www.ncbi.nlm.nih.gov/pubmed/?term=Martín-Santamaría S%5BAuthor%5D&cauthor=true&cauthor_uid=18000937).

Martín-Santamaría, *ChemMedChem* **2008**, *3*, 111-119.

^7^ N. M O'Boyle, M. Banck, C. A. James, C. Morley, T. Vandermeersch, G. R. Hutchison, *J. Cheminf.* **2011**, *3*, 33-46.
